# Bridging animal and clinical research during SARS-CoV-2 pandemic: A new-old challenge

**DOI:** 10.1016/j.ebiom.2021.103291

**Published:** 2021-04-01

**Authors:** Martin S. Winkler, Tomasz Skirecki, Frank M. Brunkhorst, Sara Cajander, Jean-Marc Cavaillon, Ricard Ferrer, Stefanie B. Flohé, Alberto García-Salido, Evangelos J. Giamarellos-Bourboulis, Massimo Girardis, Matthijs Kox, Gunnar Lachmann, Ignacio Martin-Loeches, Mihai G. Netea, Thibaud Spinetti, Joerg C. Schefold, Antoni Torres, Florian Uhle, Fabienne Venet, Sebastian Weis, André Scherag, Ignacio Rubio, Marcin F. Osuchowski

**Affiliations:** a Department of Anesthesiology, Emergency and Intensive Care Medicine, University of Göttingen, Göttingen, Robert-Koch-Str. 40, 37085 Göttingen, Germany; b Laboratory of Flow Cytometry, Centre of Postgraduate Medical Education, Warsaw, Poland; c Dept. of Anesthesiology and Intensive Care Medicine & Center for Sepsis Control and Care (CSCC), Jena University Hospital-Friedrich Schiller University, Am Klinikum 1, 07747 Jena, Germany; d Center for Clinical Studies, Jena University Hospital, 07747 Jena, Germany; e Department of Infectious Diseases, Faculty of Medicine and Health, Örebro University, Sweden; f French National Research Agency (ANR), Paris, France; g Intensive Care Department and Shock, Organ Dysfunction and Resuscitation Research Group, Vall d'Hebron University Hospital, Vall d'Hebron Barcelona Hospital Campus, Passeig Vall d'Hebron 119-129, Barcelona, 08035, Spain; h Centro de Investigación Biomedica En Red-Enfermedades Respiratorias (CibeRes, CB06/06/0028), Instituto de salud Carlos III (ISCIII), Av. de Monforte de Lemos, 5, 28029 Madrid, Spain; i Department of Trauma, Hand, and Reconstructive Surgery, University Hospital Essen, University Duisburg-Essen, Hufelandstr. 55, 45147 Essen, Germany; j Pediatric Critical Care Unit, Hospital Infantil Universitario Niño Jesús, Madrid, Spain; k 4th Department of Internal Medicine, National and Kapodistrian University of Athens, Medical School, Athens 12462, Greece; l Department of Anesthesia and Intensive Care, University Hospital of Modena, Italy; m Department of Intensive Care Medicine and Radboud Center for Infectious Diseases (RCI), Radboud University Medical Center, Geert Grooteplein Zuid 10, 6525 GA Nijmegen, The Netherlands; n Department of Anesthesiology and Operative Intensive Care Medicine (CCM, CVK), Charité – Universitätsmedizin Berlin, Augustenburger Platz 1, 13353 Berlin, Germany; o Berlin Institute of Health (BIH), Anna-Louisa-Karsch-Straße 2, 10178 Berlin, Germany; p Multidisciplinary Intensive Care Research Organization (MICRO), St. James's Hospital, James's St N, Ushers, Dublin, D03 VX82, Ireland; q Department of Internal Medicine and Radboud Center for Infectious Diseases, Radboud University Medical Center, Nijmegen, The Netherlands; r Department of Intensive Care Medicine, Inselspital, Bern University Hospital, University of Bern, Freiburgstrasse 18, 3010 Bern, Switzerland; s Pneumology Department, Respiratory Institute (ICR), Hospital Clinic of Barcelona - Institut d'Investigacions Biomèdiques August Pi i Sunyer (IDIBAPS) - University of Barcelona (UB), Spain; t Department of Anesthesiology, Heidelberg University Hospital, Im Neuenheimer Feld 110, 69120 Heidelberg, Germany; u Hospices Civils de Lyon, Immunology Laboratory, Edouard Herriot Hospital, 5 Place d'Arsonval, 69003 Lyon, France; v EA 7426 "Pathophysiology of Injury-Induced Immunosuppression - PI3", Université Claude Bernard Lyon 1/bioMérieux/Hospices Civils de Lyon, Edouard Herriot Hospital, 5 Place d'Arsonval, 69003 Lyon, France; w Institute for Infectious Disease and Infection Control, Jena University Hospital-Friedrich Schiller University, Am Klinikum 1, 07747 Jena, Germany; x Institute of Medical Statistics, Computer and Data Sciences, Jena University Hospital-Friedrich Schiller University, Bachstrasse 18, 07743 Jena, Germany; y Ludwig Boltzmann Institute for Experimental and Clinical Traumatology in the AUVA Research Center, Donaueschingenstrasse 13, 1200, Vienna, Austria

**Keywords:** COVID-19, Animal model, Pre-clinical research, Pandemic, Clinical trial, Vaccine

## Abstract

Many milestones in medical history rest on animal modeling of human diseases. The SARS-CoV-2 pandemic has evoked a tremendous investigative effort primarily centered on clinical studies. However, several animal SARS-CoV-2/COVID-19 models have been developed and pre-clinical findings aimed at supporting clinical evidence rapidly emerge. In this review, we characterize the existing animal models exposing their relevance and limitations as well as outline their utility in COVID-19 drug and vaccine development. Concurrently, we summarize the status of clinical trial research and discuss the novel tactics utilized in the largest multi-center trials aiming to accelerate generation of reliable results that may subsequently shape COVID-19 clinical treatment practices. We also highlight areas of improvement for animal studies in order to elevate their translational utility. In pandemics, to optimize the use of strained resources in a short time-frame, optimizing and strengthening the synergy between the preclinical and clinical domains is pivotal.

## 1 Introduction

In the era of COVID-19 pandemic, the spreading speed of the severe acute respiratory syndrome coronavirus type 2 (SARS-CoV-2) and coronavirus disease 2019 (COVID-19) as well as SARS-CoV-2/COVID-19-related information is unprecedented. The accumulation of scientific publications eclipses the numbers generated during any other outbreak in the last 100 years; by March 2021 >110,000 COVID-19-related publications (PubMed) and >14,000 on preprint health science servers (medRxiv, bioRxiv), e.g., compared to 114,124 all-time PubMed hits for “acute myocardial infarction”. The overall SARS-CoV-2/COVID-19 clinical information has outpaced data produced by animal-based studies (about 1:16 ratio in PubMed and 1:7 in medRxi; March 2021). This is understandable given that animal studies aiming to decipher disease pathophysiology and testing of experimental therapies typically emerge as a secondary activity. However, a rapid expansion of preclinical testing capacity may markedly enhance anti-pandemic strategies, e.g. in a form of negative screening of potential drug candidates to exclude low-impact compounds and more efficiently streamline the drug and vaccine development processes. Notably, such an expansion contradicts long-term goals set by various inter(national) policy makers (including the 2019 EU Directive (2010/63/EU) aiming at radical reduction and elimination of animal safety & drug testing [1]. Given the investigative burden of the current pandemic, it appears that animal-based research plays a central supportive role in the quest for quality scientific information. Instead of its reflexive down-scaling, a concerted effort should be made to develop routes by which reliable animal-based findings are generated - accounting both for ethical consideration and emergency needs such as the current (and imminent future) pandemic(s). Such a quality pre-clinical knowledge, when efficiently merged with the clinical domain, may facilitate a more rapid understanding of COVID-19 pathophysiology and improve patient care [2], [3], [4]. With this in mind, we present an overview of current animal and human COVID-19 studies with the goal of providing information, inspiration, and guidance to clinical and basic researchers. This review i) provides a detailed overview of the current status of both preclinical and clinical studies on COVID-19, including their strengths and limitations, and ii) aims to identify important points of synergy between these two research areas. We further propose a list of priorities that we believe are critical to the synchronization of preclinical and clinical studies and thus to a more rapid and effective response to the pandemic.

## 2 Pre-clinical models and COVID-19 phenotypes

This chapter provides a focused overview of the animal modeling landscape in the specific context of the animal-to-human research interaction discussed in the subsequent chapters. Several recent reviews provide exhaustive species-specific modeling details [5,6] and a National Institutes of Health (NIH) provide up-to-date information on animal COVID-19 models (https://opendata.ncats.nih.gov/covid19/animal).

**Fig. 1:**
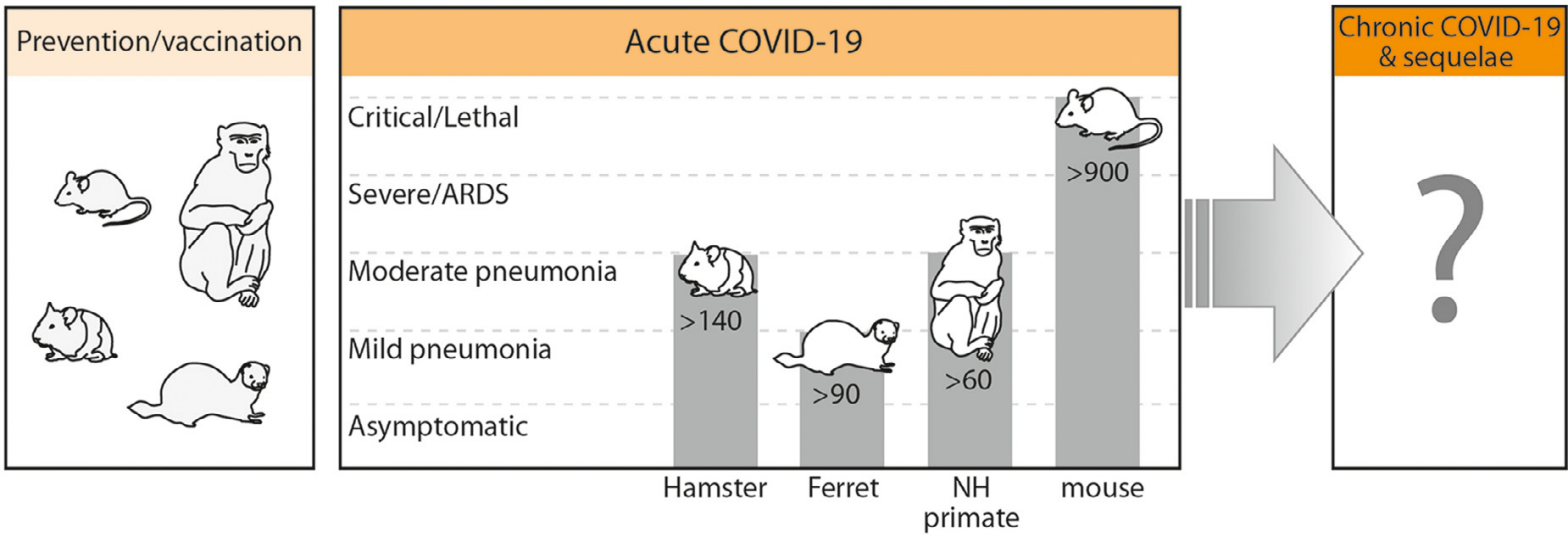
The current landscape of animal models and their utility in the SARS-CoV-2/COVID-19 pandemic. The figure includes four species currently indicated by the WHO as reproducible COVID-19 models. Vaccines in their developmental phase are commonly tested in Rhesus macaque and mice (shown); infrequently in other species such as hamster and ferret (shown) as well as rat, guinea pig, pig, sheep and others (not shown). A current list of species reported to be used for COVID-19 vaccine and drug development outside of peer-reviewed press is detailed in an interactive map of the European Animal Research Association (https://public.flourish.studio/visualisation/1698667/). To date (February 2021), hydroxychloquine, favipiravir and TLR2/6 agonist were the only peer-reviewed therapeutics preventively tested in animals (NHP, ferret, hamster). Lethal COVID-19 phenotype was reported in three studies employing the mouse model. Aged (but not young) NHP displayed a moderate pneumonia (accompanied by relatively mild clinical symptoms) presentation after infection with SARS-CoV-2. Ferrets typically displayed mild symptoms accompanied by a matching pneumonia severity. In hamsters, an advanced lung pathology can be produced but it is concurrent with mild disease symptoms. In all tested species a non-symptomatic phenotype upon infection with SARS-CoV-2 was demonstrated. No animal study focused on the chronic COVID-19 phenotype and its sequelae has been conducted yet. NH: non-humane (primate), ARDS: Acute respiratory distress syndrome. The top of each gray bar indicates the maximal severity specific for a given species; all milder phenotypes, visualized by the gray bar area, can also be produced. The number overlaid on bars depicts an approximate count of the PubMed-listed studies (prevention, vaccination and disease model) utilizing a given species (PubMed queried on February 18, 2021).

Several species were already tested regarding their utility in reproducing COVID-19 (Table 1). The World Health Organization (WHO) currently indicates four species as reproducible COVID-19 models: non-human primates (NHP), ferrets, hamsters and mice (Fig. 1). Except for mice, all indicated species are susceptible as wild-type (WT) and genetic manipulations have not been attempted. WT mice are resistant to SARS-CoV-2 infection as the viral spike protein has a minimal affinity to the murine angiotensin converting enzyme-2 (ACE2) receptor. Approaches utilized to obtain SARS-CoV-2-to-ACE2 binding in mice are chiefly based on either i) the pathogen or ii) host's manipulations. In the first, the SARS-CoV-2 either undergoes a serial passaging that introduces permissible mutations in the receptor-binding domain (RBD) of S-protein [7,8] or relies on reverse genetics modifying S-protein RBD to bind the murine ACE2 [9]. From the host's perspective, susceptibility to infection is achieved by introduction of human ACE2 gene (hACE2) [5,10]. hACE2 transgene can be either expressed under a tissue-specific promoter (*Krt18* in epithelial lineage) [11] or a non-specific highly active promoter (e.g. CMV) [12]. Additional strategies include placing the human transgene under a murine ACE2 promoter [13] or by knock-in of human cDNA in the locus of murine *Ace2* [14,15]. A transient expression of hACE2 in the murine respiratory tract can be also achieved by transduction of hACE2 expressing cassette using adenoviral [16,17] or adeno-associated virus [10]. The transient expression model may facilitate a rapid humanization in numerus mouse strains providing a powerful tool for mechanistic studies [5,10].

**Table 1:** Overview of principal pre-clinical models used to investigate COVID-19

Species	Susceptibility	Recorded Symptoms	Clinical symptoms	Lung pathology		Viral load	Specific IgG	Virus shedding via#	Airborne transmission&	Anti-COVID-19 treatment testing
				Pneumonia	Inflammatory infiltrates					
Non-human primate§ [32,33,35,53,[156], [157], [158], [159], [160]]	High, Old more susceptible	Elevated BT Decreased appetite Dehydration Irregular respiration Tachypnea Dyspnea Asthenia	mild-to-moderate	yes	yes	+++	yes	Oral/nasal/ rectal swabs, BAL, feces	N/A	• Remdesivir [53, 161] • HCQ [51] • Dalbavancin [162] • Vaccine [32,[57], [58], [59],^61^,^158^,[163], [164], [165], [166], [167], [168], [169], [170], [171], [172], [173], [174]] • Neutralizing mAb [105,175] • Catalase [56]
Ferret [25,28,[38], [39], [40],[176], [177], [178], [179], [180]]	High	Elevated BT Rhinorrhea Cough Sneezing Decreased appetite Weight loss Asthenia Decreased activity	none-to-mild	yes	yes	+++	yes	Air flow, direct contact, nasal wash saliva, urine feces, serum	Yes 10/5	• Lopinavir-ritonavir, HCQ sulfate, emtricitabine-tenofovir [38] • TLR2/6 agonist [181] • Neutralizing mAb [174] • Vaccine [176,177,180]
Hamster [30,31,36,55,[182], [183], [184], [185], [186], [187], [188], [189], [190]]	High, Old more susceptible	Tachypnoe Weight loss Asthenia Ruffled furs Intestinal inflammation	mild-to-moderate	yes	yes	+++	yes	Nasal wash, feces & N/A	Yes 38/34	• Favipiravir, HCQ/azithromycin [52] • Neutralizing mAb [55, 174, [191], [192], [193], [194], [195]] • Ranitidine bismuth citrate [196] • Vaccine [62,165,197]
Tree shrew [23,24]	Low-to-high, Age-dependent susceptibility similar	Decreased appetite Elevated BT	none-to-mild	yes	yes	Yes different tissues	Not detected	Nasal/throat, anal swabs, serum	N/A	N/A
Mouse hACE2 mouse [10,[13], [14], [15], [16], [17],^37^,^42^,^43^,^49^,^64^] WT BALB/c [8,9,31] C57BL [7, 10,31]	High, Old more susceptible	Difficulty breathing Weight loss Asthenia	mild-to-lethal*	yes	yes	+++	yes	Feces & N/A	N/A	• Pudilan xiaoyan [49] • Neutralizing mAb [17,192] • Convalescent plasma & Remdesivir [16,37,54] • Baicalein [198] • Dalbavancin [162] • interferon-λ1a [9] • Vaccine [8,9,64,165,199] • Plitidepsin [50]
Cat [25,26]	High, Young more susceptible	Asymptomatic	N/A	yes	N/A	++	Yes	Naso-pharynx, nasal wash, serum, feces	Yes 6/6	N/A
Dog [25,200,201]	Low	Asymptomatic	N/A	no	N/A	-/+	Yes, in half	Feces & N/A	No 2/0	N/A

⁎ Mortality recorded in at least five mouse studies [7,15,42,202,203]

# Both infectious and non-infectious viral shedding

& Data as: total number of naive animals exposed/number of naïve SARS-CoV-2 positive animals; animals from all studies per species combined

§ NHP include: Rhesus macaques (*Macaca mulatta*), crab*-*eating macaques (*Macaca fascicularis*), baboon (Papio hamadryas), Rivet/African green monkey (*Chlorocebus spp.*) and common marmoset (*Callithrix jacchus*). BAL: bronchoalveolar lavage; BALB/c: BALB/c mouse strain; BT: body temperature; C57BL: C57BL mouse strain; hACE2: human angiotensin converting enzyme-2; HCQ: hydroxychloquine; mAb: monoclonal antibody; mAb: N/A: not available; NHP: non-human primate; WT: wild-type.

Apart from ACE2, the newest evidence implicates additional receptors contributing to the SARS-CoV-2 tropism and cellular entry (e.g. acting as co-factors) with a potential propensity to specific tissues/cells. For example, neuropilin-1 and CD147 receptor were shown to be highly expressed in olfactory epithelial cells and brain [18,19] whereas the expression of tyrosine-protein kinase receptor UFO (AXL) was high in pulmonary and bronchial epithelial cells [20].

Less experimentally prominent but infection-permissible (also via airborne route) species include symptomatic (with pneumonia) minks [21,22] and tree shrews [23,24] as well as asymptomatic cats [25,26] and rabbits [27]. Dogs [25], pigs [25,28] and poultry [29] are generally considered SARS-CoV-2-resistant.

### 2.1 General symptoms and age effect

All WHO-recommended species (also others) robustly shed virus via the respiratory tract and present with a varying magnitude of symptoms (synopsis in Table 1). Assessment of clinical COVID-19 symptoms greatly varies among species; in NHP and medium-size species (e.g. ferrets, cats), human-like respiratory examination endpoints/techniques may be effectively utilized. In small species (hamsters, mice), due to technical constraints, such an assessment is typically less precise and based on non-respiratory parameters (e.g. asthenia, ruffled fur, hunching, weight loss); methods such as micro-CT although utilized [30,31] are less common.

NHP including cynomolgus macaques, African green monkeys and common marmosets recapitulate several features of human COVID-19 albeit with generally mild symptoms predominating (www.who.int/publications/m/item/covid-19-animal-models-summary-of-progress-made-by-the-WHO-covid-19-modelling-15-26-march-2020). Macaques, especially *Macaca mulatta* appears to be the most susceptible to SARS-CoV-2; they developed COVID-19-like lung lesions albeit with moderate symptoms [32]. Presently, aged Rhesus macaques [33,34], baboons [35], hamsters [30,36] and mice [[7], [8], [9],^14^] developed more severe disease (versus young) ranging from weight loss, exacerbated pneumonia, cellular pulmonary infiltration and cytokine release to respiratory dysfunction. Notably, one mouse study reported hyposmia/anosmia after intranasal SARS-CoV-2 inoculation [37]. Additionally, aged Rhesus [33] and tree shrews [23] displayed prolonged virus shedding whereas viral clearance was delayed in aged BALB/c mice [9] although the viral loads across organs were typically age-independent in mice [8,9,14], tree shrews [23] and higher in aged macaques [33]. Ferrets exhibit upper respiratory tract symptoms, fever and distinct lung pathology [25,[38], [39], [40]] resembling those occurring in mildly ill humans. Ferrets also display a SARS-CoV-2 shedding via multiple routes (for up to 8 days) and are susceptible to airborne infection (Table 1). Hamsters constitute another reproducible experimental option (Table 1); the infection model is stable and pneumonia is evident in the majority of infected animals. Similarly to ferrets, hamsters display a robust airborne SARS-CoV-2 transmissibility from infected to naïve animals.

### 2.2 Severity and lethality

While the majority of SARS-CoV-2 susceptible species display respiratory symptoms (including COVID-19-like pneumonia), they generally lack hypoxemic respiratory failure, extrapulmonary organ dysfunction and lethality (Table 1). Two recent studied reported advanced (but non-lethal) lung abnormalities by a micro-CT examination (accompanied by up to 20% weight loss) in SARS-CoV-2-infected hamsters [30,31], while other hamster-based studies did not report overtly severe phenotypes and lethality (Table 1). Initially, in mice infected with SARS-CoV-2, a relatively more severe disease phenotype (compared to other laboratory animals) has been reflected by up to 20% body weight loss but without mortality. Since July 2020, a lethal phenotype in murine models was reported by at least five studies (Table 1) that utilized mouse-adapted virus and various forms of ACE2 receptor humanization to facilitate the SARS-CoV-2-to-ACE2 receptor binding. Further larger studies need to precisely establish the occurrence and characteristics (e.g. early vs. late mortality) of the lethal phenotype and its causes (e.g. virus inoculum volume, application routes, pulmonary vs. extrapulmonary involvement) in those emerging mouse-based models. It appears that the general severity typically increases with age; old (surviving) macaques presented with more pronounced pneumonia concurrent with severe pulmonary lesions, inflammation and edema [33,34] and these derangements were also largely recapitulated in aged mice [7–9,14]. In hamsters, although the infection model may produce even a severe lung pathology (shown in aged subjects), mild disease symptoms predominate. Summarizing, the current evidence suggests that aged Rhesus macaques and the mice (across different ages) with an enhanced ability for SARS-CoV-2 binding most closely display the COVID-19 phenotypes observed in the intensive care unit (ICU) patients.

## 3 Current limitations in SARS-CoV-2/COVID-19 animal modeling

Animal COVID-19 models should ideally provide a full recapitulation of COVID-19 pathophysiology represented by an array of the clinical phenotypes and their sequelae and be widely available. Current high demand for experimental animals exceeds capacity of commercial breeders impeding the investigative progress and verification of the existing findings. The limited lethality across SARS-CoV-2/COVID-19 models constitutes a critical shortcoming (Fig, 1, Table 2). To maximize translational value, several key model design elements need to be contemplated: i) severe COVID-19 phenotypes (short and long-term), ii) aged animals as they correspond to the most commonly affected patient demographic, iii) the most frequent COVID-19-related comorbidities (e.g. diabetes, obesity, cardiovascular), iv) sex effects. The lack of those components fail to provide an adequate clinical outcomes match-up with the most severe COVID-19 patients. The available models can be relatively easily enriched by some elements (e.g. both sexes, comorbidities, age) that will likely induce more severe phenotypes and possibly evoke some extrapulmonary complications. The existing evidence supports this notion; SARS-CoV-2-infected mice showed viral replication and shedding in the brain [14,15,[41], [42], [43]], aged NHP [33] and male mice (also after SARS-CoV) [44] displayed a higher mortality [15] while female tree shrews demonstrated higher SARS-CoV-2 load in tissues and swabs [23].

**Table 2:** Limitations and solutions in animal modeling of SARS-CoV-2/COVID-19

COVID-19 specific modeling limitations:	Improvement possible?	Possible action(s)
Lack of mortality and/or severe COVID-19 phenotypes	yes	employing higher viral load/aged animals/comorbidities remodeling interactions between ACE2-SARS-CoV-2 (e.g. serial passage, reverse genetics) immuno-suppression induction (constant, transient)
Absence of systemic endotheliitis	unclear	-“- & using genetically modified animals with propensity for endothelial inflammation & hypercoagulation models with chronic endothelial pathology (e.g. atherosclerosis)
Lack of comorbidities*	yes	including any relevant co-morbidity
rodent ACE2 fail to bind SARS-CoV-2	yes	humanizing mouse ACE2, ACE2-SARS-CoV-2 adaptation/remodeling
Limited extra-pulmonary symptoms/derangements	unclear	higher viral load/aged animals/comorbidities, remodeling interactions between ACE2-SARS-CoV-2

Non-specific modeling limitations:	Improvement possible?	Possible action(s)

Limited use of mature/aged animals	yes	inclusion of aged animals
Limited critical care-like protocols	with difficulty	advanced ICU-like setups (e.g. ventilation, central lines)
Absence of long-term monitoring	yes	protracted monitoring in animals with severe phenotypes to test for long-term effects
Lack of comorbidities^#^	yes	including any relevant co-morbidity
Anatomical/physiological differences	with difficulty	confirmatory studies across various species, humanized animals
Interspecies immune systems differences	limited	expression of human genes, transplantation of human cells
Restrictive legislation for critical care models	yes	cooperation with ethical and regulatory bodies

⁎ comorbidities including pulmonary illnesses, diabetes, hypertension, obesity that predispose for severe COVID-19 phenotypes, # any other pre-existing conditions and genetic alterations that are not currently known to predispose/protect against severe COVID-19 phenotypes

Other shortcomings are difficult and impossible to overcome. Immuno-modulatory agents against COVID-19 are difficult to investigate given different inter-species immune responses. To improve mechanistic understanding, a given therapeutic should preferably be tested in different species. Furthermore, while the availability and ease of use of experimental animals are inversely correlated to their size, the physiological and anatomical similarities are not. For example, the lethal phenotype and neurological sequelae position the mouse as a translationally-attractive model for COVID-19. Unfortunately, the mouse is an inferior model of human airway diseases given its e.g., monopodial (versus human trichotomous) airway branching system, much smaller alveolar and ventilatory unit diameter, richer bronchus-associated lymphoid tissue and differences in regenerative pulmonary response [45], [46], [47]. A reflexive over-reliance on a single-species disease model may produce misleading findings that are rapidly propagated until contradicted in other species. This limitation is aggravated with the rare pre-clinical use of ventilatory support - the hallmark of severe COVID-19 treatment. While technically possible, its complexity practically eliminates mechanical ventilation from large-scale animal studies [48]. The work with SARS-CoV-2 virus is further burdened by the enhanced biosafety (BSL-3) requirements (increasing costs and limiting modeling capacity) and increasingly restrictive ethical consideration (especially in the EU countries) regarding the use of animals models at the highest severity classification [1].

## 4 Animal models to test anti-COVID-19 therapies

The emergence of the first reproducible models that partly recapitulate COVID-19 has enabled limited *in-vivo* testing. The first study testing a traditional Chinese medicine in hACE2 mice was published on May 5, 2020 [49], followed by studies examining the efficacy of several antivirals, chloroquine (CQ), hydroxychloquine (HCQ), neutralizing monoclonal antibodies (mAb) and convalescent plasma (Table 1 provides a complete list of treatment studies against SARS-CoV-2/COVID-19 per listed species). A ferret study demonstrated a potential utility of emtricitabine-tenofovir in reducing COVID-19 symptoms but inefficacy of lopinavir-ritonavir [38]. Plitidepsin that undergoes clinical testing (NCT04382066), reduced SARS-CoV-2 infection and COVID-19 lung pathology in mice [50]. While in vivo findings should be interpreted with caution given the infancy of the COVID-19 modeling, the emerging data are largely compatible with the findings from the largest clinical trials. For example, CQ/HCQ demonstrated neither preventive nor therapeutic benefit in non-critical pneumonia in NHP [51], ferrets [52] and hamsters [52]. However, remdesivir demonstrated some potential benefit in NHP [53] and mice [16,54] and the same holds true for convalescent plasma and neutralizing monoclonal Abs tested (pre-and post-infection) in mice [16,17,37] and hamsters [55] (Table 1). Given the current clinical uncertainty regarding the latter therapeutics, animal data should not be reflexively dismissed as unreliable but further explored to gain knowledge why they were successful and utilize this information in optimization of subsequent human trials including endpoints, cohort selection, qualitative antibody enrichment, application routes. A recent pharmacokinetic COVID-19 study demonstrated an advantage of a nebulization route for nanocapsuled catalase over its systemic delivery in Rhesus macaque [56]. Pre-clinical evidence rarely translates 1:1 to clinical evidence; the fidelity of animal studies should not be judged on the anticipated results but rather the quality of their unbiased and robust design involving various species.

## 5 Utility of animal models in vaccine development

In February 2021, the WHO reported 63 candidate vaccines in clinical trials and 179 in preclinical evaluation (https://www.who.int/publications/m/item/draft-landscape-of-covid-19-candidate-vaccines). Twenty vaccines entered phase 3 trials and demonstrated interim benefits have led to regulatory approval/authorization of 10 vaccines https://www.nytimes.com/interactive/2020/science/coronavirus-vaccine-tracker.html). The development process of COVID-19 vaccines, including some of the most advanced Janssen-Cilag [32], Sinovac [57], Oxford/AstraZeneca [58], Moderna [59], Pfizer/BioNTech [60] and Sinopharm [61] heavily depended on animal testing (Table 3). The key pre-clinical step typically involved vaccine efficacy testing in Rhesus macaques. In four of those studies, immunization with the vaccine candidates was also performed in BALB/c (Sinovac, Oxford/AstraZeneca, Pfizer/BioNTech, Sinopharm), CD1 mice (Oxford/AstraZeneca) and in Wistar (Sinovac) and Sprague Dawley rats (Sinopharm). Rhesus macaques have been generally preferred given that they become clinically symptomatic following the SARS-CoV-2 challenge. Although a SARS-CoV-2 infection in young macaques cannot be considered a severe COVID-19 disease model, non-vaccinated animals developed pulmonary pathology resembling clinical presentation [[57], [58], [59],^61^]. Of note, the adenovirus vaccine (Johnson & Johnson) tested in Rhesus macaques produced a complete protection in 17 while partial/non-protecting response in 22 animals [32]. In hamsters, vaccination attenuated pneumonia and weight loss post-SARS-CoV-2 infection [62,63]. Furthermore, adapting the SARS-CoV-2 virus (S-protein RBD remodeling and serial passaging) [8,9] and genetic modifications (hACE2 mice) [64] aiming at exacerbating the mouse COVID-19 severity markedly expand the potential for vaccine testing.

**Table 3:** The use of animal SARS-CoV-2 infection models in vaccine development*

Species & strains	Intervention	Endpoints
Inbred BALB/c mice (1,3,5,6)	Immunization with vaccine candidate	Antibody titers (1,3,5,6), T- & B-cell counts (5), T-cell responses (3,5). Inflammation & other (nonspecified) adverse events (1).
Outbred CD1 mice (3)	Immunization with vaccine candidate	Antibody titers, T-cell responses
Wistar rats (1) Sprague-Dawley rats (6)	Immunization with vaccine candidate	Antibody titers Inflammation & other (nonspecified) adverse events (6)
Rhesus macaques (1,2,3,4,5,6)	Immunization with vaccine candidate followed by challenge with SARS-CoV-2 (1-6).	Post-immunization effects: Antibody titers (1,2,3,4,5), T-cell counts (1), T-cell responses (2,3,4,5), plasma cytokines (1,3), BAL immunophenotyping (4), biochemistry (1), histopathology of various organs (1), fever (1), weight loss (1), appetite (1), mental state (1), non-specific adverse events (3). Post-challenge effects: viral load in respiratory tract and lungs (1–6), bronchoalveolar fluid cytokines (2), BAL immunophenotyping (4), pulmonary histopathology (1,2,3), respiratory tract histopathology (3), histopathology of other organs (3), clinical score (3), nonspecified clinical signs of disease (5,6)

1: Sinovac [57]; 2: Moderna [59]; 3: Oxford/AstraZeneca [58] 4: Janssen-Cilag [32]; 5: Pfizer/BioNTech [60]; 6: Sinopharm [61]

BAL: bronchoalveolar lavage

⁎ Studies on vaccine candidates in an advanced stage (regulatory approval granted/pending or phase III trials near completion or completed) are included.

Previous animal studies alerted to relevant immunopathology induced by several vaccine candidates against SARS-CoV [65,66] and research on the Dengue virus vaccine [67] indicated that application of S-protein specific antibodies can enhance viral replication (so-called antibody-dependent enhancement; ADE) and skew the macrophage response upon SARS-CoV infection in mice and NHP [68,69]. The existing SARS-CoV-2 studies mitigate these concerns; in none of the tested species, any serious adverse events were observed post-immunization [57,58]. Moreover, in vaccinated macaques, the SARS-CoV-2-induced inflammatory response and histopathological changes were attenuated compared to non-vaccinated controls. Given the emergency need, it has been postulated by some to omit animal-based safety testing and to directly transition vaccine candidates to phase-1 trials; some companies have partly followed that fast-track route [70]. Such an accelerated development, however, is considered very controversial by many experts [70,71] as it risks exposing human recipients to: i) potential adverse effects and ii) to non-toxic but no/low-efficacy vaccines. To increase a possibility of detecting any adverse effects during the preclinical safety testing, it is advisable to employ more than a single species.

## 6 Clinical trials in the pandemic

According to registry data (https://covid19.trialstracker.net), the increase of clinical studies since the onset of the pandemic is unprecedented. As of March 2021, there were over 6400 entries on COVID-19 clinical studies. The following section provides a summary of the currently active/recruiting phase II-IV trials testing immuno-modulating substances, which subjectively constitute: i) the largest and most dynamic therapeutic domain, and ii) the most challenging area for animal-to-patient translation and iii) it remains unclear whether hypo- or hyperinflammation responses dominate in COVID-19 progression. The subsequent sections discuss key challenges related to the design and execution of clinical trials.

### 6.1 Corticosteroids

Four randomized clinical trials (RCTs; including RECOVERY) indicated a potential clinical benefit of intravenous dexamethasone/hydrocortisone in critical COVID-19 patients [72], [73], [74], [75]. In the CoDex study enrolling 299 patients with moderate-to-severe ARDS, intravenous dexamethasone increased ventilator-free days (*verum* 6.6 days, control 4.0 days) [74]. However, little is known about potential side-effects and the benefit–risk profile of corticosteroids across the full spectrum of COVID-19 patients [76]. Although RECOVERY did not report adverse events, corticosteroids are potent immunosuppressants which may impair viral clearance [77], increase secondary infections (fungal, bacterial) and promote metabolic adverse events [78]. Additionally, targeted corticosteroid delivery via inhalation is investigated in six ongoing trials (NCT04355637; NCT04416399; NCT04331470; NCT04377711 & NCT04193878; NCT04330586). Inhaled corticosteroids (for obstructive pulmonary disease) were associated with an increased risk of upper respiratory tract infection, and there is scarce evidence of benefit of inhaled corticosteroids in COVID-19 [79,80]. Interestingly, asthma patients treated with inhaled corticosteroids appear to present a reduced sputum cell expression of ACE-2 and transmembrane protease serine 2 (required for cellular entry by SARS-CoV-2) [81]. In a recent Korean study, a prior use of inhaled corticosteroids did not increase COVID-19-related mortality [82]. Summarizing, time-limited corticosteroid therapy (approx. 10 treatment days) is beneficial in selected critically ill cases. Additional data on exact timing, dose, administration route, and potential side effects (e.g. immunosuppression, secondary infections) is required to optimize the corticosteroid treatment guidelines.

### 6.2 Cytokines, chemokines and their receptors as targets

Multiple trials evaluate modulation of diverse cytokines: either as application of the recombinant protein, such as granulocyte-macrophage colony-stimulating factor (GM-CSF; three trials), interferon (IFN)-α (one trial), the neutralization of cytokines such as macrophage colony-stimulating factor (M-CSF; one trial), interleukin (IL)-6 (nine trials), IL-1β (one trial), tumor necrosis factor (TNF) (one trial), TNF superfamily member 14 (TNFSF14; one trial), and their receptors such as GM-CSF receptor (one trial), IL-1Ra (eight trials), and IL-6R (one trial). Few studies target chemokines e.g. IL-8 (one trial) or chemokine receptors, such as CCR5 (six trials). The majority of these trials use humanized antibodies and few trials employ small molecule-based inhibitors.

Immunotherapies directed at pro-inflammatory cytokines were suggested as adjunctive treatment in COVID-19. While smaller studies provided variable results with anti-IL-6 (siltuximab) and anti-IL-6 receptor (tocilizumab) antibodies [83], the recent REMAP-CAP showed an improved outcome in the ICU COVID-19 patients treated with tocilizumab [84] and the newest press release by RECOVERY trial announced its life-improving efficacy in hospitalized COVID-19 patients (www.recoverytrial.net). Similarly, the off-label use of recombinant human IL-1Ra (anakinra) in 52 COVID-19 patients was associated with reduced ICU admission for mechanical ventilation and death [85]. However, anakinra was not beneficial in mild-to-moderate COVID-19 [86]. Conversely, the anti-IL-1β antibody canakinumab did not influence outcome in 450 COVID-19 patients (NCT04362813). As anakinra blocks the biological activity of both IL-1α and IL-1β, whereas canakinumab inhibits only IL-1β, this may suggest that the effectiveness of anakinra is attributable to the IL-1α blockade. A multicenter SAVE-MORE anakinra trial stratifying COVID-19 patients based on the soluble urokinase plasminogen activator receptor concentration may provide a deeper therapeutic insight (NCT04680949). Other cytokine targets include TNF (monoclonal antibody – infliximab – and a recombinant fusion TNF receptor - etanercept) and IFN-γ (emapalumab, a monoclonal antibody). Baricitinib is a selective Janus kinase (JAK1/JAK2) blocker that inhibits the gp130 family cytokines (e.g. IL-6,-12,-23 and IFN-γ); it can potentially inhibit viral infection of cells by inhibiting kinases involved in viral endocytosis [87]. GM-CSF is considered a multifaceted contributor to the COVID-19 pathogenesis with both administration and inhibition currently studied [88]. Summarizing, there have been numerous attempts to limit the consequences of a “cytokine storm” (CS) even though it remains unclear whether a CS phenomenon in COVID-19 truly exists and to what extent it might contribute to the disease pathophysiology (Lancet RD in press). The variable effects of anti-IL-6/IL-1 trials underline the need for well-designed cohort-based studies to pinpoint the potential therapeutic benefit of those anti-inflammatory approaches.

### 6.3 Receptor-driven signaling pathways

Several trials target downstream signaling cascades of receptors of cytokines, chemokines and pathogen recognition receptors: 24 trials test the inhibition of the JAK pathway (Ruxolitinib), six trials of inhibitors of the Bruton's tyrosine kinase, two trials of sphingosine kinase 2 (Opaganib) and one trial inhibition of phosphoinositide 3-kinase. Two studies target pattern recognition receptors toll like receptors (TLRs) 2/6/9 and cytosolic NLRP3, the receptor component of the canonical inflammasome complex. Regarding T-cells, divergent approaches aim at either boosting the cellular activity with Thymosin-1α (four trials) and anti-program cell death protein (PD)-1 antibody (three trials) and inhibiting its activation with cyclosporine (10 trials) and IMU-838 (an inhibitor of dihydroorotatedehydrogenase of activated lymphocytes; five trials). 71 trials evaluate the application of recombinant interferons, alone or in combination with other drugs, with IFN-α (17 trials) and β (33 trials) to boost the T cell activity. Summarizing, based on the emerging positive results in patients with a milder COVID-19 [89,90] and evidence on the disturbed interferon responses in the blood compartiment, interferon-replenishing interventions appear to belong to the most promising modulators.

### 6.4 Cell-based therapies

Cell based therapies such as intravenously and intrapulmonarily delivered mesenchymal stem cells (MSC) have gained interest as a potential treatment in COVID-19-related lung injury given their anti-inflammatory and immunoregulatory abilities with possible effects on the lung tissue repair. Presently, three published phase I trials investigating MSCs in severe COVID-19 showed promise [91], [92], [93]. These single-site Chinese studies demonstrated that MSC infusion was safe for up to 28 days. However, due to the study-design limitations the true therapeutic gains cannot be defined.

Overall, 55 clinical trials investigating cell-based therapies are currently underway (phase I n = 12, phase I/II n = 22, phase II n = 15, phase II/III n = 5, phase III n = 1), using MSCs of different origins (e.g., placental, adipose tissue-derived MSCs) and isolated SARS-CoV-2 specific T cells. Furthermore, native natural killer (NK) cells (4 trials) and genetically modified NKG2D-ACE2 CAR-NK cells (1 trial) are tested; both strategies aim at recognizing/inactivating virus-infected ACE2-expressing cells. To increase efficacy and reduce the cytokine release syndrome (CRS) risk, those genetically engineered NK-cells are modified to secrete both GM-CSF neutralizing single-chain variable fragment (scFv) and an IL-15 agonist (sIL-15/IL-15Rɑ chimeric protein) inhibiting the otherwise short NK-cell survival time. Summarizing, there is no evidence currently supporting the use of cell-based therapies in COVID-19. To evaluate survival and tissue repair effects of cell-based therapies in COVID-19, randomized phase 2/3 trials with a long-term follow up and relevant mechanism-of-action endpoints are required.

### 6.5 Other immunomodulators

The use of nonsteroidal anti-inflammatory drugs in COVID-19 patients is deliberated [94]. Early therapy with ibuprofen as a cyclooxygenase inhibitor was hypothesized beneficial in preventing disease progression and reversing lymphocytopenia [95] and a large trial enrolling patients with mild-to-moderate respiratory failure is underway (NCT04334629). Intravenous and inhaled iloprost (an endothelium-protective prostaglandin analogue) are under evaluation in two randomized trials in patients undergoing mechanical ventilation [96]NCT04445246). The anti-complement (C)-5 monoclonal antibody Eculizumab and the anti-C3 cyclic peptides AMY-101 have been used in COVID-19 [97] and clinical trials are underway (NCT04395456; NCT04333420). Moreover, the effects in reducing lung injury and promoting repair mechanisms of complement C5 inhibitor Zilucoplan is the aim of a small study in COVID-19 patients with moderate-to-severe ARDS [98]. In another small-scale trial, 28-day mortality decreased following (C5a-targeting) vilomemlimab treatment [99]. Intravenous polyclonal immunoglobulins therapy combined with steroids improved hypoxia and reduced hospital length-of-stay in severe COVID-19 patients [100]; an ongoing multicenter RCT will verify these effects. Several non-randomized trials using different blood purification methods reported benefits in COVID-19; subsequent ongoing trials evaluate the appropriate timing, dosing and methodology [101]. Summarising, immune dysregulation and ineffective immuno-inflammatory responses are frequently observed in response to SARS-CoV-2 infection. The use of immunomodulators has a potential to at least partly remedy these derangements. However, their safety and impact on the development/progress of COVID-19 across patients groups remain unclear and caution is advised.

### 6.6 SARS-CoV-2 neutralizing antibodies

In animals, there has been some evidence that neutralizing antibodies (NAbs) are protective against SARS-CoV-2 infection generating an interest in their potential prophylactic/therapeutic use against COVID-19. The available NAbs primarily block the glycoprotein that mediates binding to the RBD of the host ACE2 (RBD) [102]. A recently isolated S309 antibody was shown to potently neutralize SARS-CoV-2 and SARS-CoV-2/SARS-CoV pseudoviruses by engaging the RBD of the S glycoprotein [103]. Several trials evaluate the convalescent plasma and anti-spike antibodies (LY-CoV555, REGN-COV2, JS016, TY027, CT-P59, BRII-196, BRII-198 and SCTA01) to neutralize SARS-CoV-2 attachment and transmission [104]. A novel double, long-lasting monoclonal antibody (mAb) AZD7442 (derived from convalescent COVID-19 patients) currently undergoes testing (NCT04625972); its alleged advantage rests on combining two mAbs that simultaneously bind to two non-overlapping sites of the virus spike glycoprotein thus more effectively blocking the SARS-CoV-2/ACE2 interaction [105]. A critical aspect in a successful use of NAbs is their administration dose. An interim analysis of a phase 2 trial using LY-CoV555 NAb demonstrated that only the medium dose appeared to accelerate the natural decline in the SARS-CoV-2 load in outpatients with mild-to-moderate COVID-19 [106]. Summarizing, SARS-CoV-2 neutralizing antibody-based therapies possess a useful double-benefit potential: they can be applied both therapeutically and prophylactically.

## 7 Challenges in planning clinical trials in a pandemic

Previous pandemics outlined the urgent need for evidence to guide the best treatments. However, pandemics should not simultaneously serve as an excuse for lowering scientific standards [107]. Based on statements of the REMAP-CAP investigators (https://www.remapcap.org) [108], there are several organizational challenges in the planning/launching of clinical COVID-19 studies given that the onset and/or epidemiological dynamics of a pandemic are unpredictable [109,110]*.* To at least partly remedy such challenges, the WHO advised developing “Master Protocols”, especially for high-powered, multi-center trials to support the generation of high-quality data (https://www.who.int/blueprint/priority-diseases/key-action/novel-coronavirus/en/).

One of the key complexities is that the precise nature of the infecting pathogen(s), clinical consequences and suitable interventions (particularly pathogen-specific) are not known in advance and inferring information from previous pandemics of related origins can be misleading and dangerous [111]. In the context of SARS-CoV-2/COVID-19, this accounts for a poorly understood period of the clinical progression (i.e. from an initial infection to life-threatening respiratory infection). It is likely that a proportion of patients presented with community-acquired pneumonia may feature a less severe phenotype, yet remain at risk of rapidly progressing to severe illness. Patients who require hospital admission but have a less severe illness are a particularly important group: an early intervention at a medium-severity illness may prevent progression to the life-threatening phenotype. Additionally, the proposed treatments will likely elicit differential treatment effects depending on the magnitude of the illness severity at the time of treatment administration. A range of scenarios must be anticipated and used to provide guidance regarding the most appropriate/beneficial response.

### 7.1 Time-critical generation of evidence

A typical pandemic elicits a high number of cases occurring over a short period (weeks, months). In the early phase of the COVID-19 pandemic, researchers heavily relied on so-called “real world” data e.g. from sources such as electronic medical records, patient registries, reimbursement/claims data or published single center case series. All of these have an advantage of an *ad hoc* availability with a trade-off of data/analytical quality [112] that may ultimately lead to false conclusions and/or recommendations (e.g. HCQ). Inappropriate evaluation of the "real world" data caused confusion regarding the alleged preventive benefits of chronic oral anticoagulants [113,114]; a careful methodological insight revealed analytical shortcomings (e.g. non-comparable research questions, uncertainties of the estimators).

When more traditional clinical trials are considered, one or few research questions are typically the driver of a prospectively designed study with a study protocol. The pandemic has taught us that such trials are possible and lead to valid conclusions. However, we have also learned that they have to be pragmatic - often large and simple - and only work with an established technical and organizational infrastructure. The latter includes appropriate responses from ethical committees and the public as well as hospital authorities. The often cited major difference between trials that rely on Frequentist *vs.* Bayesian statistics are of lesser importance compared to these points [115]. Table 4 summarizes large randomized controlled trials that evaluate/evaluated several different treatments in COVID-19 patients.

**Table 4:** The largest multi-center clinical trials

Clinical trial	Interventions/Study arms	Target population	Outcomes	Adaptive design
RECOVERY NCT04381936 Participants estimated: 20000	Lopinavir-Ritonavir: stopped Low-dose dexamethasone [Ref. 1]: stopped Hydroxychloroquine: stopped Azithromycin: ongoing Convalescent plasma: ongoing Tocilizumab: ongoing Immunoglobulin: ongoing Synthetic neutralizing antibodies: ongoing Aspirin: ongoing Colchicine: ongoing	Adults Hospitalized patients Critically ill patients (suspected/confirmed COVID-19)	Reduced mortality by 30% in critically patients, and by 20% in hospitalized patients Reduced duration of hospitalization Reduced progression to MV and CRRT	YES
REMAP-CAP NCT02735707 Participants estimated: 7100	Lopinavir/ritonavir: ongoing Hydroxychloroquine: ongoing Hydroxychloroquine + lopinavir/ritonavir: ongoing Interferon-β1a: ongoing Anakinra: ongoing Fixed-duration higher dose Hydrocortisone: Stopped Tocilizumab: ongoing Sarilumab: ongoing Vitamin C: ongoing Therapeutic anticoagulation: ongoing Simvastatin: ongoing Convalescent plasma: ongoing Protocolised mechanical ventilation strategy: ongoing Eritoran: ongoing Apremilast: ongoing Aspirin: ongoing Clopidogrel: ongoing Prasugrel: ongoing Ticagrelor: ongoing	Adults Critically ill patients receiving invasive or non-invasive respiratory support, or vasopressors (probable/confirmed COVID-19)	21-day survival (days alive and not receiving organ support in ICU)	YES
SOLIDARITY NCT04647669 Participants recruited: 11330	Remdesivir: stopped Hydroxychloroquine: Stopped Lopinavir/ritonavir: Stopped Acalabrutinib (anti-tyrosine kinase): ongoing Interferon β1a: Stopped	Hospitalized adults	28-day mortality All 4 treatments evaluated (remdesivir, hydroxychloroquine, lopinavir/ritonavir and interferon) had little or no effect on overall mortality, initiation of ventilation and duration of hospital stay in hospitalized patients.	YES
PRINCIPLE ISRCTN86534580 Participants estimated: 3000	Azithromycin: ongoing Doxycycline: ongoing Inhaled budesonide: ongoing	Primary care Outpatients	The risk of complications from suspected COVID-19	YES
DISCOVERY NCT04315948 Participants estimated: 3100	Remdesivir: ongoing Lopinavir/ritonavir: ongoing Interferon Beta-1a: ongoing Hydroxychloroquine: ongoing Standard of care: ongoing	Hospitalized and critically ill adults (Confirmed SARS-CoV-2 infection)	Percentage of subjects reporting each severity rating on a 7-point ordinal scale (at day 15): Not hospitalized, no limitations on activities Not hospitalized, limitation on activities; Hospitalized, not requiring supplemental oxygen; Hospitalized, requiring supplemental oxygen; Hospitalized, on non-invasive ventilation or high flow oxygen devices; Hospitalized, on invasive mechanical ventilation or ECMO Death	YES

CRRT: Continuous renal replacement therapy; ICU: Intensive care unit; MV: mechanical ventilation

## 8 Interaction between clinical and animal research

Design of animal research for translational purposes should be largely aimed at supporting the preparation and execution of clinical trials. This should be done by using the entire available pre-clinical armamentarium including pathomechanistic investigations, preselection of therapeutics, identification of non-and responder subgroups, identification and optimization of endpoints. To achieve desired synergy, such a process requires knowledge on the limitations and advantages of a given model and intricacies of proper study design that maximally synchronizes with the stringent demands of the clinical-level research. Especially in a pandemic, such converging areas should be developed given the pressing need for a rapid understanding of COVID-19 and improvement of patient outcomes. Below, we present investigative areas with the strongest potential for enhancement of the animal-to-human investigative synergy.

### 8.1 COVID-19 severity

Both mild and severe COVID-19 phenotypes are instrumental for adequate pre-clinical testing of preventive and therapeutic strategies. The currently predominating mild-to-moderate model phenotype should not be reflexively rejected as inadequate, however, given that it enables detection of any adverse effects of tested substances. By employing high lethality of given disease models, a frequent design feature in critical illnesses, such a design is primarily set toward observing hypothesized benefits rather than undesired complications [116,117]. In the current pandemic, a massive number of trial-tested substances has been registered - a vast majority with a relatively low patient enrollment. Drug pre-testing performed in mild-to-moderate COVID-19 models has a strong potential of detecting i) the lack of efficacy in non-critically ill and ii) undesired adverse effects, thus sparing patients from unnecessary exposure to ineffective and/or potentially harmful substances and directing them toward more promising interventions. HCQ constitutes a prime example (a detailed coverage of HCQ controversy in COVID-19 elsewhere [118]); its premature COVID-19 efficacy-hype was recently denied by the high-quality trials and all animal tests performed to date [38,51,52]. In this context, the already available COVID-19 models could be rapidly employed to investigate potential adverse effects of corticosteroids - the only recommended anti-COVID-19 treatment. Animal pre-testing is also relevant as the current costly deluge of trials overwhelms the medical staff, may cause competition for COVID-19 patients and promotes inferior and/or unethical trial design practices [119], [120]. Once reproducible, severe COVID-19 models are available, we suggest a multivariate testing across mild, moderate and severe phenotypes as it has the best potential to advise on the clinical trial decision-making.

### 8.2 Cohort targeting

Compared to human trials, animal studies have a strong advantage regarding pre-selection features. Comparative testing can be relatively easily executed in divergent homogeneous cohorts displaying a desired pre-defined characteristic, e.g. male/female, young/old, early/late, lung/gastro-intestinal phenotype. In trials, these elements are typically assessed retrospectively (thus with weaker cause-effect relationship powers). Some treatments may elicit contrasting effects depending on the COVID-19 characteristics and/or type of targeted patients; a defined pre-clinical cohort-testing will likely provide an informational groundwork for clinical investigations. Animal cohort studies can be i) performed before a trial is launched, ii) run parallel to an ongoing trial to support testing of defined hypotheses (qualified as impossible and/or unethical in trials) and/or iii) rapidly executed to confirm/refute observations from ongoing and finished trials. The emerging evidence underlines the importance of cohort-oriented research; aged patients are at risk for unfavorable outcomes whereas females are less prone to the most severe COVID-19 phenotypes [121]. Such cohort comparisons are relatively easy to reproduce in animals and frequently match clinical observations. For example, female mice demonstrated superior resistance to infectious respiratory diseases [44,122] while male mice displayed a more pronounced mortality after SARS-CoV infection [44]. Additionally, transgenic animals can effectively address the role of comorbidities. For example, the effects of diabetes/obesity and their consequences on COVID-19 can be studied in genetically-manipulated mouse models [123].

### 8.3 Pathophysiological insight

Relevant animal models, either wild-type or genetically manipulated, appear indispensable for investigation of specific SARS-CoV-2-induced pathomechanisms such as hypercoagulability and immuno-inflammatory responses (e.g. compartmentalization) associated with various disease phenotypes. The explorative possibilities should not be limited to molecular research but also assess physiological responses (such as respiratory failure [124,125]. Lessons from other critical care models (e.g. sepsis) must be learnt to avoid simplistic approaches, e.g. acute care treatments (e.g. fluid resuscitation, antimicrobials) [126,127] and prospective risk stratification tools [128] should be routinely employed. COVID-19 models must also feature disease-characteristic comorbidities to maximally reflect the pathomechanisms occurring in patients e.g. obesity-influenza [129], asthma-influenza [130], stress-sepsis [131]. Such an approach can better i) elucidate causative relationships between the infection and a given comorbidity and ii) explore their underlying mechanisms. COVID-19 models with comorbidities may likely develop more severe/lethal phenotypes and long-term impairments (e.g. lung fibrosis, fatigue, cognitive dysfunctions). Introduction of wild-type microbiome and pathobiome into mice could enable investigation of host-microbiome-SARS-COV-2 interactions [132]. Another refinement are two-hit models; they typically involve two (simultaneous, sequential) acute conditions (e.g. trauma/hemorrhage, secondary infections) and are superior (versus single-hit) in reproducing complex responses recorded in acute patients [133]. In COVID-19, two-hit models could effectively recapitulate frequent scenarios of critically-ill patients developing secondary infections (e.g. hospital-related pneumonia, fungemia). Several two-hit models helped to unravel pathways responsible for secondary infection-triggered pathology and mortality [134] and disclosed training-priming effects to protect against aggressive infections [135,136].

### 8.4 Standardization

Standardization of COVID-19 models will enhance reliability and trust in preclinical findings. While translation of animal data to the clinic is not ideal, numerous examples of successful replications of preclinical findings in acute clinical care exist [137,138] including medications [139] and treatment criteria [140]. Modeling standardization has been already successfully implemented in diabetes [141,142], stroke [143], epilepsy [144] and sepsis [145]. An even more advanced improvement would be a creation of international collaborative COVID-19 preclinical research platforms that simultaneously investigate the most relevant targets/hypotheses. Operation Brain Trauma Therapy is an example of such a multicenter consortium (drug and biomarker screening) in traumatic brain injury [146]. A coordinated multicenter approach is especially advisable for NHP studies; individual NHP experiments are poorly powered and already a two-center study increases the power of size treatment estimate by approximately 40% without a need for larger sample size [147]. Pre-clinical data obtained from standardized SARS-CoV-2/COVID-19 studies (based on blinding, randomization and multi-center approach) will be superior in enhancing their utility to guide clinical decisions on i) aborting/entering/progressing of experimental substances in clinical trials, ii) directing focus to specific patient cohorts for both benefits and harm, iii) elucidating specific pathophysiological phenomena, and others.

### 8.5 Investigating emerging SARS-CoV-2 mutations

It was initially assumed that vaccines would likely match all circulating variants [148], however, the newest non-peer reviewed findings on SARS-CoV-2 mutations question that assumption. New variants emerging in Denmark (mink-human infections) with the D796H and ∆H69/V70 spike mutations resulted in a markedly lower sensitivity/recognition of the virus by convalescent patient sera [149]. The deletion ∆H69/V70 is also present in the rapidly spreading UK variant; it shares the mutation N501Y with the South-African and the Brazilian variants and the experimentally mouse-adapted SARS-CoV-2 virus [7]. The two latter variants and the Japanese variant share the E484K mutation that appear to render them more infective and partly resistant to neutralizing antibodies (preprinted studies: [150], [151], [152]). Emergence of the new SARS-CoV-2 variants creates a potential investigative niche for animal models; despite obvious interspecies differences, they could be utilized to test whether the emerging mutations result in an altered replication, transmissibility, infectiousness and severity of the infection [153], [154], [155]. Interestingly, the serial lung-based passage of SARS-CoV2 generated a mouse-adapted strain bearing N501Y mutation resembling the above-mentioned human variants [8]. Immunized animals can also serve as a rapid verification platform for vaccination efficacy, i.e., whether or not the acquired immunity following the use of approved and/or emerging vaccines remains protective independently of the mutations. This is particularly important, because the consequences of the mutations were mainly analyzed regarding the reactivity of antibodies from convalescent plasma and vaccinated subjects while the T-cell immunity and/or global protective immunity were not assessed.

## 9 Synthesis

COVID-19 pandemic has revealed the limitations of rapidly designed studies and confusion caused by unreliable data. Yet, a medical threat of this magnitude requires immediate actions. Given this incongruity, any proposed priorities must balance the need for a rapid but high-quality response.

### 9.1 Challenges in animal research

A rapid development of informative animal models appear crucial in any pandemic. However, such a process should not equal a rampant generation of publishable but low-quality models.

Priority 1: Rapid and consensus model selection. A major focus should be on a rapid consensus selection and use of high-quality modeling studies reflecting the largest possible array of clinical disease scenarios and that are relatively easily reproducible by specialized pre-clinical research centers. Pre-clinical work with any highly infectious pathogen causes various technical impediments and only a limited number of centers (academic, non-profit, commercial) are capable of overcoming them. Such impediments delay any developmental process and limit possible options.

Priority 2: Promotion of expert networks and data sharing. It appears beneficial to promote establishment of pre-clinical networks of expert-consortia with resources enabling a rapid launch of research whenever required. Such consortia should combine academic, non-profit and for-profit research centers and be authorized to rapidly and consensually share information. A formalized collaborative format will exploit available synergies and lead to development of rigorous and reproducible animal models, which can be then quickly implemented for investigative purposes.

Priority 3: Safeguarding stable funding and ethical approval. Given the fact that existing expert consortia may best allow for timely launch of animal model studies, a consensus public funding and ethical approval (across the entire spectrum of severity) should be rapidly obtainable for respective expert-institutions. We propose that the respective national government funding agencies and ethical committees have consensually prepared contingency plans allowing a rapid and synchronized launch of the designated pre-clinical consortia.

### 9.2 Challenges in clinical research

While multiple potential biological targets may be known (or inferred), classic drug development is delayed in a fluctuating pandemic and the presence of virulent pathogens. A global medical crisis limits implementation of state-of-the-art designs and not all available drug candidates can be simultaneously tested.

Priority 1. Judicious selection of therapeutics for initial testing*.* It is most efficient to use existing, approved substances whose efficacy, risks are known from other indications, and from which a therapeutic benefit in COVID-19 (or any future emergency disease) can be assumed. Experimental substances may potentially offer excellent treatment benefits but should be viewed as secondary testing choices due to a protracted safety/efficacy testing process and high risk of failure.

Priority 2. Reliance on high-quality multicenter pragmatic RCTs. Most of the available SARS-CoV-2/COVID-19 evidence stems from low-quality studies. This diverse non-RCT data segment, although attractive given its simple design and relatively rapid data generation, typically provides unreliable findings that may provoke erroneous changes in treatment guidelines. High-quality RCTs should be preferred given that despite delay, they provide the most reliable evidence of treatment efficacy, based on which lasting treatment recommendations can be formed.

Priority 3: Separation of patients based on their phenotypic characteristics. COVID-19 has multifaceted presentation and accurate characterization of various disease phenotypes is central for selection of adequate therapies (e.g. with immunomodulators). Such a cohort selection allows i) precise trial enrollment of defined patient types and ii) reverse-translation of the specific subgroups into respective animal cohorts for further characterization and testing of therapeutics. Previous trial failures (e.g. in sepsis) imply that such phenotypic separation is pivotal for the development of individualized interventions.

## 10 Outstanding questions

Using all available tools, scientists and physicians share an accountability to understand pathophysiology of the diseases they combat to maximize the efficacy of treatments and minimize adverse effects. In the translational research context, the current pandemic provokes several questions aimed at operationalization of the above-formulated priorities:

1 How to most effectively facilitate (fiscally, ethically) and streamline the development of animal models so they achieve a maximal fidelity to the clinical COVID-19 presentations?
2 Which elements of the already known evidence should be prioritized for bidirectional verification between animal and clinical studies to enhance understanding of COVID-19 pathophysiology?
3 How to organize, coordinate and optimize global research-exchange networks amalgamating both pre-clinical and clinical evidence in a pandemic?
4 How to effectively promote and facilitate preference for advanced multi-center RCTs over proliferating low-quality trials?

## 11 Search strategy and selection criteria

First, panels of 2–3 authors, selected based on their expertise, created individual review chapters/sections/tables. Second, each draft was critically reviewed by all other authors. Third, all authors revised their drafts based on the received feedback and carried out a final update to include all most recent relevant studies published during the writing of the manuscript. The first and corresponding authors conducted the final assembly of the reviewed chapters. To identify the relevant information, the authors searched PubMed and/or Medline for articles published in English by December 2020, using a panel of search terms and/or combinations thereof, always including one of the following terms: "SARS-CoV-2", "COVID-19", "MERS-CoV", “SARS-CoV”, "SARS-CoV-1", "coronavirus". Search of medRxiv/bioRxiv was performed to identify and monitor progress of potentially relevant publications but referencing to publications without peer-review was kept to minimum and only when necessary (e.g. in the *Testing of emerging SARS-CoV-2 mutations* section).

## 12 Contributors

TS, JMC, MK, SW, GL, IML, SC, FU and MFO wrote the Animal Modeling-related sections; FMB, EJGB, SBF, MGN, MG, AS, SC, FV, RF and AGS wrote the Clinical Trials-related sections; IR, TS, MSW and MFO wrote the Animal-Human Interactions section; MFO, MSW, TS, AT, JCS and IR wrote Introduction, Synthesis and Outstanding Questions. Additionally, IR, MW and MFO created Fig. 1; MFO, TS, JMC, TS and GL created Table 1; MFO, MSW, TS and IR created Table 2; MK, SW created Table 3, RF, FMB and AS created Table 4. All authors took part in conceptualization of the review, data curation, critical review of the entire manuscript including the Fig. 1 and Table 1, Table 2, Table 3, Table 4, final revision and data curation update to include all most recent relevant studies published during the writing of the manuscript. MSW, TS, IR and MFO conducted the final assembly and editing of all chapters. All authors read and approved the final version of the manuscript.

## Declaration of Competing Interest

The authors declare no conflicts of interest.
